# Persistent Suppression of Type 1 Diabetes by a Multicomponent Vaccine Containing a Cholera Toxin B Subunit-Autoantigen Fusion Protein and Complete Freund's Adjuvant

**DOI:** 10.1155/2013/578786

**Published:** 2013-11-11

**Authors:** Béla Dénes, István Fodor, William H. R. Langridge

**Affiliations:** ^1^Center for Health Disparities and Molecular Medicine, Department of Biochemistry, School of Medicine, Loma Linda University, 11085 Campus Street, Loma Linda, CA 92350, USA; ^2^Department of Immunology, Central Veterinary Institute, Tábornok u. 2, 1143 Budapest, Hungary

## Abstract

Data presented here demonstrate multifunctional vaccination strategies that harness vaccinia virus mediated delivery of a gene encoding an immunoenhanced diabetes autoantigen in combination with complete Freund's adjuvant (CFA) that can maintain safe and durable immunologic homeostasis in NOD mice. Systemic coinoculation of prediabetic mice with recombinant vaccinia virus rVV-CTB::GAD and undiluted or 10-fold diluted CFA demonstrated a significant decrease in hyperglycemia and pancreatic islet inflammation in comparison with control animals during 17–61 and 17–105 weeks of age, respectively. Synergy in these beneficial effects was observed during 43–61 and 61–105 wks of age, respectively. Inflammatory cytokine and chemokine levels in GAD-stimulated splenocytes isolated from vaccinated mice were generally lower than those detected in unvaccinated mice. The overall health and humoral immune responses of the vaccinated animals remained normal throughout the duration of the experiments.

## 1. Introduction

Type 1 diabetes mellitus (T1D) is a chronic metabolic disease that is based on autoimmunity and is most frequently initiated in childhood. Initial symptoms include autoreactive lymphocyte mediated progressive destruction of the insulin-producing beta islet cells of the pancreas triggered by the innate and ultimately the adaptive arm of the body's immune system. This early perturbation of immunological homeostasis results in a progressive loss of islet *β*-cell function, leading to an overall insulin deficiency and resulting in elevated blood sugar levels (hyperglycemia), increased cellular oxidative stress leading to chronic pancreatic islet inflammation, and an associated risk for development of secondary neural and circulatory health problems, resulting in amputation of the extremities, blindness, and increased probability of heart attack and stroke [1]. Type 1 diabetes incidence is steadily increasing in the western world [2]. In the United States, approximately 3 million Americans are afflicted with all forms of diabetes, of which from 15 to 20% currently suffer from T1D. Showing the extensive nature of this disease, more than 13,000 children are diagnosed with T1D in the U.S. annually. Hyperglycemia, the major manifestation of clinical diabetes, represents the final outcome of immunological processes that have progressed over a number of months in mice and years in humans. Treatments for disease prevention which focus on arresting or reversing hyperglycemia are inadequate, as islet *β*-cell destruction is completely asymptomatic until more than half of the approximately 1 × 10^6^ islets in the human pancreas have been irreversibly inactivated or destroyed. Familial inheritance studies show that genetic factors play a significant role in T1D development, and at least 15 genetic loci have been linked to T1D susceptibility in the nonobese diabetic (NOD) mouse model [3]. However, since type 1 diabetes occurs in only approximately 50% of monozygotic twins, genetic risk factors are insufficient to account for disease occurrence [4]. Environmental factors, including virus infection and dietary components, are thought to contribute to diabetes onset [5, 6]. Following CD4^+^ autoreactive T helper (Th) cell infiltration of pancreatic islets in NOD mice, autoreactive effector Th1 lymphocytes were shown to secrete inflammatory cytokines IFN-*γ* and IL-2. These cytokines stimulate macrophage and CTL secretion of oxidative compounds and inflammatory cytokines that induce chronic pancreatic inflammation (insulitis) and were shown to contribute to the apoptosis of islet insulin-producing *β*-cells [3]. A variety of immune cells including dendritic cells, macrophages, natural killer (NK) cells, and B cells have also been shown to participate in diabetes pathogenesis [5]. B cells influence the developing autoimmune T-cell response mainly in the initial stages of T1D development [7]. At present, there is no established clinical approach that can effectively suppress long-term T1D. However, a sufficient number of *β*-cells may exist at the time of diagnosis of T1D, and diabetes could be reversed once the autoimmune response is rapidly suppressed. Recent studies show that the development of a vaccine that prevents autoreactivity or reestablishes immune regulation once autoreactivity occurs may provide a promising therapy for T1D treatment. A list of the major autoantigens in T1D includes insulin, glutamic acid decarboxylase (GAD), insulinoma antigen (IA-2), and several other islet beta cell proteins [8]. In general, two strategies of molecular vaccination have been developed: viral vector-based and recombinant plasmids, both carrying genes for autoantigens, and/or immunomodulatory proteins. In our earlier studies, partial diabetes suppression was observed following vaccinia virus (rVV-CTB::GAD) mediated mucosal or intraperitoneal inoculation of NOD mice with CTB::GAD fusion protein [9]. Recently we demonstrated that a combination of rVV-CTB::GAD with the rVV-IL10 virus expressing the antiinflammatory cytokine IL-10 was effective in preventing diabetes onset in NOD mice [10]. Complete Freund's adjuvant (CFA) containing heat-killed mycobacteria (*M. tuberculosis*) demonstrated beneficial effects in the prevention of diabetes onset in NOD mice [11]. While the underlying mechanism remains unknown, CFA may act in part by enhancing the ability of NOD mouse antigen presenting cells activation of NOD CD4^+^ CD25^+^ regulatory cells responsible for the control of autoreactive T-cells and prevention of disease in NOD mice [12]. However, the level of immune system stimulation from normally inoculated doses of CFA may cause unacceptable side effects. Therefore, here we investigate whether virus-delivered CTB::GAD treatment in combination with reduced CFA dosages can provide effective, durable, and safe prevention in prediabetic NOD mice.

## 2. Materials and Methods

### 2.1. Multicomponent Vaccine Construction

The vaccinia virus construct rVV-CTB::GAD used in this study contains a cDNA fragment encoding the diabetes pancreatic islet autoantigen GAD55, made up of a truncated form of human GAD65 minus the N-terminal membrane binding region (aa 89–585) linked to the C-terminus of the cholera toxin B subunit gene (CTB), as previously described in [9]. Complete Freund's adjuvant (CFA) was purchased from Sigma-Aldrich Co. (St. Louis, Mo). Each mL of adjuvant contained 1 mg of *Mycobacterium tuberculosis* (H37Ra, ATCC 25177), heat killed and dried, 0.85 mL paraffin oil, and 0.15 mL mannide monooleate. CFA was diluted 1 : 10 and 1 : 100 in phosphate-buffered saline (PBS).

### 2.2. Detection of Hyperglycemia in Immunized NOD Mice

Four-week-old female NOD LtJ mice were purchased from Jackson Laboratory (Bar Harbor, ME) and maintained in the animal care facility of the Central Veterinary Institute, Budapest, Hungary. The protocol for mouse rVV inoculation was approved by the Animal Research and Care Committees of Loma Linda University School of Medicine (Loma Linda, CA) and the Central Veterinary Institute in Hungary. Prior to measurement of hyperglycemia, a total of eight experimental groups of 5-week-old NOD mice (*n* = 10) were subjected to subcutaneous (s.c.) injection with 0.1 mL of undiluted, 1 : 10 and 1 : 100 diluted CFA, or/and intraperitoneal (i.p.) inoculation with 0.3 mL rVV-CTB::GAD (5 × 10^7^ PFU/mL). Two weeks after the first injection, the vaccine inoculations were repeated. One mock-infected experimental group (*n* = 10) was inoculated with PBS only.

The mice were fed complete mouse chow and water *ad libitum*. To detect the onset of hyperglycemia, beginning at 13 wks of age, the mice in each experimental group (Table 1) were bled from the tail vein biweekly and blood sugar levels were quantified. Diabetes was confirmed when blood glucose levels exceeded 14 mmol/L for two consecutive weeks. Blood glucose levels were confirmed with Keto-Diastix urinary glucose test strips (Bayer AG, Leverkusen, Germany). Experiment was completed at 105 wks of age. After that, in selected experimental groups, indications related to aging (tumors, liver degenaration, and heart problems) were observed. The incidence of hyperglycemia was considered to be insignificant between experimental groups when the calculated *Z* value was between −1.96 and +1.96. The Mann-Whitney *U* test was also applied to compare hyperglycemia incidence and blood glucose levels among groups. Statistical significance was determined at *P* < 0.05. A synergistic effect in the combined two-component treatment (CTB::GAD + CFA) was considered when each component alone (rVV-CTB::GAD or CFA) did not show a statistically significant difference compared to naive mice, while the combined treatment (rVV-CTB::GAD + CFA) did result in a statistically significant difference in comparison with naive mice. Additive effect is observed when both components alone, as well as combined treatments result in statistically significant differences compared to naive mice. The difference in effects conferred by naive and combined two-component-treated mice is larger than that conferred by each component alone.

### 2.3. Histopathological Analysis and Computer-Assisted Morphometry Measurement of Pancreatic Islets

In this study pancreatic islets of hyperglycemic and euglycemic mice were analyzed separately. Mice that developed blood glucose concentrations of 33 mmol/L (hyperglycemic) were sacrificed and the extent of lymphocyte islet infiltration was evaluated, as previously described in [10]. Mice that did not develop hyperglycemia over the course of the experiment (euglycemic mice) were sacrificed for histopathological analyses at 119 wks of age. The degree of insulitis was measured in each mouse based on the extent of lymphocyte infiltration of the islets. The percentage of the infiltrated area was measured using AxioVision 4 microscope software (Carl Zeiss Inc., Jena, Germany). Insulitis scores were based on a 5-level semiquantitative scale ranging from 0 to 4, where an insulitis score of 0 was considered to be normal regarding islet morphology, with no indication of autoreactive lymphocyte infiltration. Insulitis scores of 1-2 indicated progressively increasing levels of peri-islet insulitis and scores of 3-4 indicated progressive levels of intraislet insulitis, with a score of 4 equivalent to complete invasion of the islet by autoreactive lymphocytes [9].

### 2.4. Analyses of Secreted Cytokines/Chemokines and T-Cell Subsets of Splenocytes

The mice were sacrificed by CO_2_ asphyxiation and the spleens immediately excised. The splenocytes were isolated, as described in [10]. Briefly, the spleens were frozen in 90% FBS and in 10% DMSO solution and were stored at −196°C until examination. Prior to testing, frozen splenocyte samples were thawed rapidly by warming in a 37°C water bath, diluted with 25 mL of RPMI 1640 containing 10% FCS, collected by centrifugation at 250 g for 10 min, and suspended in 10 mL RPMI 1640 with 10% FCS. The splenocyte samples were then transferred into 25 cm^2^ tissue culture flask with vented cap (Sarstedt, Inc., Newton, NC) and incubated at 37°C overnight. On the next day, 1 × 10^7^ cells/mL splenocytes of the mice group naive, CFA (1 : 0), rVV-CTB::GAD + CFA (1 : 0), and rVV-CTB::GAD + CFA (1 : 10), were stimulated with GAD65 peptide (30 *μ*g/mL) comprised of amino acids 530 to 543 of the protein (AnaSpec, Inc., Fremont, CA), or without the GAD peptide (data not shown) as a control. Stimulation was performed in 15 mL polypropylene tubes held at an angle of 5 degrees for 48 h at 37°C in a humidified atmosphere of 5% CO_2_ in air. Following incubation, the splenocyte preparations were centrifuged at 350 g for 10 min at room temperature to sediment the cells. The supernatant culture medium was collected and stored at −80°C until examination for secreted cytokine content. The splenocytes were used immediately for the flow-cytometric analysis of the T-cell subsets.

For examination of CD3^+^, CD4^+^, and CD8^+^ surface markers, splenocytes were stimulated (or not in the controls, data not shown) with the GAD65. Cells then were suspended in 2 mL of PBS and centrifuged at 300 g for 5 min at room temperature. The sedimented cells were suspended again in an appropriate volume of PBS (1 × 10^7^ cells/mL). Splenocytes were stained with a three-color reagent—Mouse T Lymphocyte Subset Antibody Cocktail, with Isotype Control (BD Pharmingen, San Jose, CA), designed to identify major subsets of T lymphocytes by direct immunofluorescent staining with flow cytometric analysis. This cocktail consisted of a mixture of PE-Cy7 hamster anti-mouse CD3e, PE rat anti-mouse CD4, and FITC rat anti-mouse CD8a antibodies. An equivalent concentration of fluorochrome- and isotype-matched negative-control immunoglobulins was used as the Mouse T Lymphocyte Subset Isotype Control. Subsequently, the samples were vortexed and incubated for 30 min at room temperature in the dark, and then the cells were centrifuged at 300 g for 5 min at room temperature. After removing the supernatant, the sedimented cells were suspended in 2 mL of BD CellWASH solution (BD Biosciences, San Jose, CA) followed by centrifugation at 300 g for 5 minutes at room temperature. The supernatants were removed and cells were fixed by 0.5 mL of CellFIX solution and flowcytometry was performed using a BD FACSCalibur flow cytometer (BD Biosciences). The one-way Anova was used to evaluate the statistical significance of differences in the percentage of positive cells. Experimental values were considered significant at *P* < 0.05.

The supernatants of the splenocytes stimulated with GAD65 peptide of mice inoculated with PBS, CFA (1 : 0), rVV-CTB::GAD + CFA (1 : 0), and rVV-CTB::GAD + CFA (1 : 10), were used for the analyses of cytokine/chemokine secretion. The relative levels of selected mouse cytokines and chemokines were determined using Proteome Profiler Array, Mouse Cytokine Array Panel A kit (R&D Systems, Inc., Minneapolis, MN) according to the manufacturer's instruction. Briefly, the membranes were transferred into a 4-well multidish and were blocked in Array Buffer 6 by incubation for one hour on a rocking platform. One mL of the pooled supernatant samples from each group was transferred to 0.5 mL of Array Buffer 4 in separate tubes. Reconstituted Mouse Cytokine Array Panel A Detection Antibody Cocktail (15 *μ*L) was added to each prepared sample. The samples were mixed and incubated at room temperature for one hour. After removing the Array Buffer 6 from the 4-well multidish, the sample/antibody mixtures were added to the membrane and were incubated overnight at 2–8°C on a rocking platform shaker. Following the incubation, each membrane was washed two times with a 1x wash buffer for 10 minutes on a rocking platform shaker. Two mL of diluted in Array Buffer 6 Streptavidin-HRP were pipetted into each well of the 4-well multidish, and then the membranes were returned to the Multi-dish and were incubated for 30 minutes at room temperature on a rocking platform shaker. Following the incubation, the membranes were washed as described above, and then 1 mL of the prepared Chemi Reagent Mix was added evenly onto each membrane. The membranes were placed in an autoradiography film cassette, and were exposed to X-ray film for 1, 5, and 10 minutes. Pixel densities on developed X-ray film were collected and analyzed using a transmission-mode scanner and image analysis AxioVision 4 software.

### 2.5. Detection of IL-10 and IL-12 in NOD Mouse Serum

The BD Mouse IL-10 Flex Set and the BD CBA Mouse IL-12p70 Flex Set Bead Based Immunoassay were used to measure IL-10 and IL-12 levels in serum samples, respectively. The sets were used in conjunction with a BD CBA Mouse/Rat Soluble Protein Master Buffer kit, a BD FACSCalibur flow cytometer, and the FCAP array software according to the manufacturer's instructions (all from BD Biosciences). In brief, 50 *μ*L of the mixed capture beads was transferred into each assay tube. Serum samples (50 *μ*L) were diluted 1 : 4 in assay diluent and transferred to the assay tubes. The tubes were incubated in the dark for 2 h at room temperature. Following the incubation, a mixed PE detection reagent was added to each assay tube (50 *μ*L/test) and incubated in the dark for 1 h at room temperature. The samples were washed with 1.0 mL of wash buffer followed by centrifugation at 200 g for 5 minutes. The supernatant was removed and 300 *μ*L of Wash Buffer was added to the assay tubes and was briefly vortexed before analyzing samples by flow cytometry. In addition, a serial dilution of the BD CBA Mouse or Rat Soluble Protein Flex Set Standard was used to establish a standard curve for accurate determination of the secreted cytokine levels in each sample. The Student's *t*-test was used to evaluate the statistical significance of differences in the serum cytokine levels. Experimental values were considered significant at *P* < 0.05.

### 2.6. VV-Specific Antibody Induction

To optimize the enzyme-linked immunosorbent assay (ELISA), a VV-specific monoclonal antibody (3B10/G9/B7) against VV A33R gene product (unpublished) was used as a positive control (not shown). Serum of naive Balb/c mice was used as a negative control. The working dilution of the VV-antigen was determined by titration in carbonate buffer (pH 9.6), NaOH (pH 13), 0.1 M Glycin (pH 2.7), and PBS (pH 7.2). To measure virus-specific IgG by ELISA, the antigen was diluted with NaOH (pH 13), and then 100 *μ*L aliquots were measured into the wells of the ELISA plate (Analyzer Ltd., Budapest, Hungary). After incubation at +4°C overnight, the plates were washed five times with PBS washing-diluting buffer containing 0.05% Tween 20 (Sigma Aldrich Co., St. Louis, MO), and then 100 *μ*L volumes of the sera diluted 1 : 5 with PBS-Tween 20 buffer were measured in the wells. The plates were incubated at +37°C for 60 min and then washed five times with PBS-Tween 20 buffer. Subsequently, 200 *μ*L rabbit anti-mouse IgG (H+L) horseradish peroxidase (HRP) conjugate (Jackson Immuno Research Labs Inc., West Grove, PA) diluted 1 : 2000 in PBS-Tween 20 buffer was measured in the wells. After a 60 min incubation at +37°C, the plates were washed as described above, and then the enzyme activity was visualized by the addition of 100 *μ*L of tetramethylbenzidine (TMB) (Diavet Ltd., Budapest, Hungary). After a 20 min incubation at room temperature, the colored reaction was stopped by addition of 50 *μ*L of 2 N H_2_SO_4_ solution per well. Optical density (OD) of samples was measured at 450 nm in a Multiscan Ms reader spectrophotometer (Labsystems Oy, Helsinki, Finland). In addition, each assay plate also contained a positive and negative control as described above. The Mann-Whitney *U* test was used to evaluate the statistical significance of differences in the optical density values. Experimental values were considered significant at *P* < 0.05.

## 3. Results

### 3.1. Suppression of Hyperglycemia in NOD Mice

**Figure 1:**
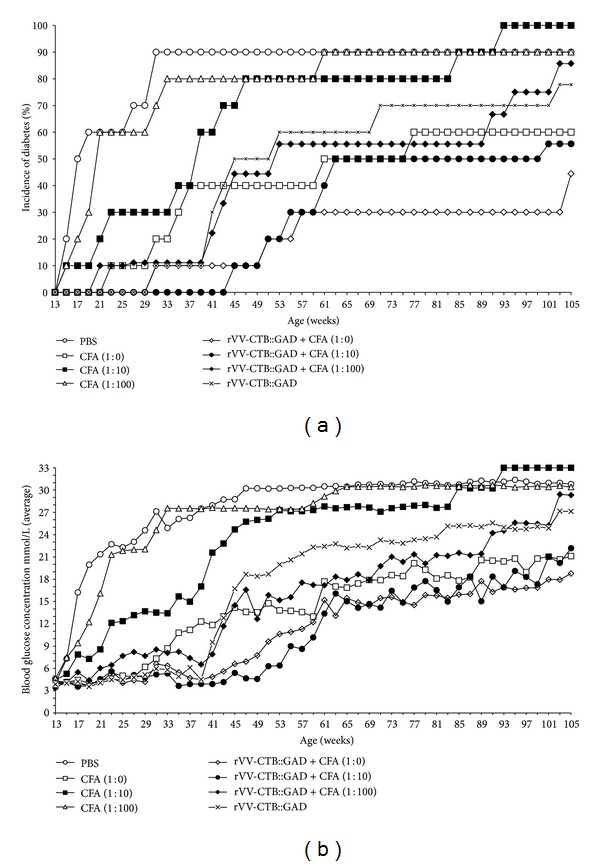
Incidence of diabetes in nonobese diabetic (NOD) mice. (a) Comparison of immunological suppression protocols on diabetes onset in NOD mice as determined by increased incidence of hyperglycemia. Treatment with rVV-CTB::GAD alone and coinoculation with CFA demonstrated statistically significant differences (*P* < 0.01–0.001) compared to the phosphate-buffered saline (PBS) (mock-infected) control group. Differences between CFA (1 : 10), CFA (1 : 100), and PBS treatment groups are statistically insignificant (Mann-Whitney *U* test). (b) Blood glucose levels in all experimental animal groups were monitored biweekly. Each data point represents the average blood glucose determination for all mice in that group until 105 weeks of age. Starting from 107 weeks of age several mice have died from unknown and unrelated to diabetes reasons; the figure does not show data points between 107 and 119 weeks. By the end of the experiment, highest blood glucose levels were detected in naive mice inoculated with PBS, CFA (1 : 10), and CFA (1 : 100), reaching average levels of 31, 33, and 30 mmol/L, respectively. Lowest levels of blood glucose maintenance were found in NOD mice inoculated with rVV-CTB::GAD + CFA (1 : 0) (18.8 mmol/L), CFA (1 : 0) (21.1 mmol/L), and rVV-CTB::GAD + CFA (1 : 10) (22.2 mmol/L). Differences in the average blood glucose levels in all groups were statistically significant (*P* < 0.05–0.001) compared to naive controls, except group of mice inoculated with CFA (1 : 100) (Mann-Whitney *U* test).

Undiluted (1 : 0) and 10- and 100-fold diluted CFA adjuvant was used in NOD mouse inoculation studies in combination with the recombinant virus rVV-CTB::GAD to assess the efficacy of the multifunctional vaccine in autoantigen-dependent enhancement of immune suppression of T1D symptoms of hyperglycemia and insulitis. Following inoculation of NOD mice with the CFA alone, the frequency of hyperglycemia in the mouse experimental groups increased at statistically significant rates depending on the nature of the treatment. The incidence of diabetes diagnosed in the control PBS group and in the CFA 1 : 100 experimental group rapidly increased from 13 wks until 31 and 33 weeks of age, respectively, reaching 90% and 80% in diabetes incidence, respectively (Figure 1(a)). At termination of the experiment, 9 of 10 PBS- or CFA 1 : 100-inoculated mice (90%) developed terminal diabetes at 31 and 61 wks, of age, respectively.

These experimental data indicate that the lowest dose of CFA (1 : 100) alone had no measurable effect on suppression hyperglycemia in the vaccinated mice. The 1 : 10 dilution of CFA alone generated an intermediate level of diabetes protection during 19 and 31–37 wks of age as statistically confirmed by “test for equality of two proportions,” in comparison with the naive control group (PBS) (Figure 1(a) and Table 2). Mice in this group gradually attained 80% hyperglycemia, which stimulated morbidity by 47 wks and 100% hyperglycemia, which stimulated morbidity by 93 wks of age. Inoculation with undiluted (1 : 0) CFA produced partial protection during 17–59 wks of age (*P* < 0.01), in comparison with the naive group (PBS), with a gradual increase in hyperglycemia to 40% by 37 wks of age and reaching a final level of 60% morbidity by 77 wks of age (Figure 1(a) and Table 2). Thus, both undiluted 1 : 0- and 1 : 10-diluted CFAs alone exert a protective effect on NOD mouse development of diabetes.

Inoculation with rVV-CTB::GAD alone provided a measurable level of protection similar to that of undiluted CFA (Figure 1(a)). The protective effects of the vaccine in this experimental group were measurable until 39 wks of age. Following 39 wks, the incidence of diabetes gradually increased to 70% at 71 wks of age. As expected, addition of CFA at a dilution of 1 : 100 did not provide improvement in CTB::GAD-mediated immune suppression of hyperglycemia. However, the combination of rVV-CTB::GAD with CFA diluted 1 : 10 substantially delayed hyperglycemia onset until 43 wks of age, after which animal morbidity gradually increased to 50% at 63 wks of age. In comparison with the control group (PBS), significant differences were measurable from 17 through 61 wks of age (Table 2). The lowest levels of hyperglycemia onset were observed following inoculation of the mice with CTB::GAD + undiluted CFA (1 : 0). Complete vaccine protection of the experimental animals was detected from 13 through 29 wks of age. However, from 29 to 57 wks of age, the level of hyperglycemia gradually increased to 30%, a level which was maintained until 103 wks of age. Statistically significant differences between the vaccinated experimental animal groups and the PBS experimental group were observed over the entire duration of the experiment (105 wks, see Table 2). Synergy in beneficial effects following treatment with the two vaccine components was observed following inoculation with rVV-CTB::GAD + CFA (1 : 10)—from 43 through 61 wks of age. Following vaccination with rVV-CTB::GAD + CFA (1 : 0) synergistic immunosuppressive effects were observed from 61 through 105 wks of age. The additive immunological suppression was observed for the rVV-CTB::GAD + CFA (1 : 10) experimental group between 17 and 43 wks of age, while for the rVV-CTB::GAD + CFA (1 : 0) treatment group the effect was observed from 17 through 59 weeks of age.

Data on blood glucose levels of various treatment groups of NOD mice are presented in Figure 1(b). Elevated glucose levels (averaging 7.32 mmol/L) were detected in the PBS control and CFA diluted 1 : 100 alone groups of mice as early as 15 wks of age. In mice inoculated with CFA 1 : 10 alone, similar blood sugar levels (7.87 mmol/L) were detected two weeks later, at 17 wks of age. However, in the following weeks, mice in this group developed hyperglycemia at a retarded level in comparison with CFA (1 : 100). The experimental group of mice inoculated with undiluted CFA alone showed increased blood sugar levels starting at 23 wks of age. However, in the experimental animal group coinoculated with CTB::GAD + CFA (1 : 0 or 1 : 10 dilution), elevated blood sugar levels were not detected until 31 and 43 wks of age, respectively (see statistical analyses in Table 2). Among mice inoculated with CTB::GAD, elevated blood sugar levels were detected as early as 29 wks of age.

**Figure 2:**
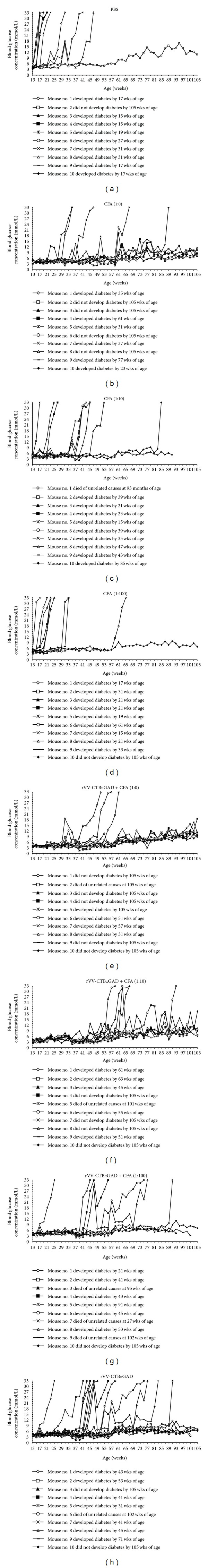
Blood glucose levels in individual animals of all experimental groups. (a) Mock-infected experimental group of 5-week-old NOD mice (*n* = 10) was inoculated with PBS. (b), (c), and (d) Three experimental groups of mice (*n* = 10) were subjected to s.c. injection with 0.1 mL of undiluted (1 : 0) and 1 : 10 and 1 : 100 diluted CFA. (e), (f), and (g) Groups of mice rVV-CTB::GAD + CFA (1 : 0), and rVV-CTB::GAD + CFA (1 : 10), rVV-CTB::GAD + CFA (1 : 100) (*n* = 10) were injected with the 0.3 mL virus (5 × 10^7^ PFU/mL) i.p. and s.c. coinoculated with 0.1 mL CFA. (h) The rVV-CTB::GAD group of mice (*n* = 10) was subjected to i.p. injection with 0.3 mL of the virus (5 × 10^7^ PFU/mL). Two weeks after the first injection, the vaccine inoculations were repeated. Beginning at 13 wks of age the individual mice in each experimental group were bled from the tail vein biweekly for 119 wks, and blood sugar levels were quantified.

**Figure 3:**
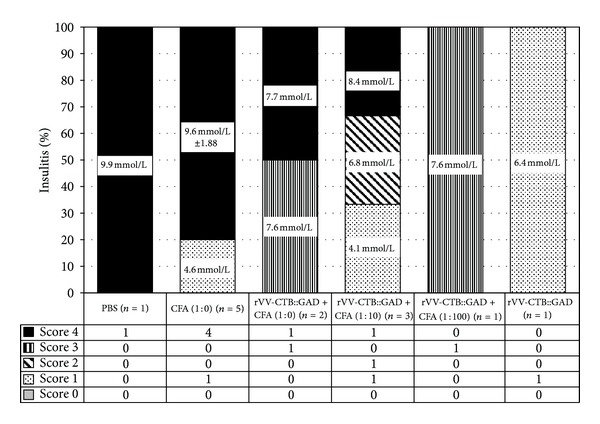
Histological analysis of insulitis in mice at the end of the hyperglycemia study. Data on pancreatic tissue cross-sections of euglycemic mice of different treatment groups obtained from mice at 119 weeks of age are shown. Altogether, 13 mice (presented in the table below the graph) were analyzed and scored for insulitis: one mouse of the PBS control, rVV-CTB::GAD + CFA (1 : 100), and rVV-CTB::GAD treatment groups, five mice of the CFA (1 : 0), three mice of the rVV-CTB::GAD + CFA (1 : 10), and two mice of the rVV-CTB::GAD + CFA (1 : 0) groups. Blood glucose concentrations of individual (in mmol/L) or grouped (mean ± SD, in mmol/L) mice are also presented.

Analyses of individual animals in all treatment and control groups are shown in Figure 2. In the control group (Figure 2(a)), only one mouse (no. 2) remained diabetes-free until the end of the experiment, showing an increase in blood sugar levels only after 71 weeks of age. However, clinical diabetes was not confirmed by urinary glucose testing (Figure 2(a)). The remaining nine mice developed diabetes indicating a relatively low level of genetic variability in experimental NOD mice. Six of nine mice became diseased by 19 wks, while the remaining three mice became hyperglycemic at 27 (no. 6) and 31 (no. 7-8) wks. Among mice inoculated with CFA 1 : 10 or CFA 1 : 100 alone, the first sick animals were detected as early as 15 wks of age. Otherwise, the pattern observed in the CFA (1 : 100) group (Figure 2(d)) was similar to that of the PBS group with the only difference being that the last mouse in this group developed diabetes with a significant delay, at 61 wks of age. From these data we can confirm the experimental results presented above indicating that the highly diluted CFA (1 : 100) alone has no detectable therapeutic effect. In contrast, the low dose of CFA (1 : 10) and undiluted CFA (1 : 0) were shown to provide beneficial effects in the prevention of diabetes onset, especially in the case of undiluted CFA (Figure 2(b)). By 21 weeks of age, none of the total 10 mice had developed diabetes. Most of the intermediate-dose CFA (1 : 10) treated mice (8 out of 10) gradually developed diabetes from 15 to 47 weeks of age (Figure 2(c)). Similarly, a moderately beneficial effect was observed in rVV-CTB::GAD and rVV-CTB::GAD + CFA (1 : 100) groups (Figures 2(g) and 2(h)). Interestingly, all cases of morbidity within the rVV-CTB::GAD + CFA (1 : 0) group, except for no. 5 (105 wks), fall between 31 and 57 weeks of age. Among mice treated with rVV-CTB::GAD + CFA (1 : 10), five animals developed diabetes at a later time, between 45 and 63 weeks of age (Figures 2(e) and 2(f)) as expected. All the mice analyzed that developed high blood glucose levels also generated a high intraislet insulitis of score = 4 (data not shown). Of 13 euglycemic mice analyzed, 4 mice (30.8%) developed peri-islet insulitis with scores of 1 and 2, and 9 mice (69.2%) developed intraislet insulitis with scores of 3 and 4 (Figure 3).

**Figure 4:**
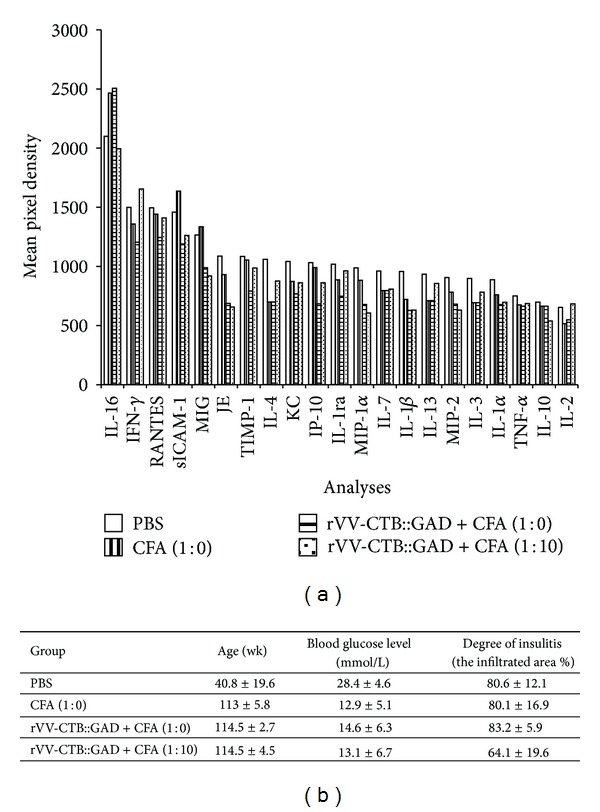
Analyses of secreted cytokines/chemokines. (a) Cytokines/chemikines synthesized by splenocytes of CFA (1 : 0), rVV-CTB::GAD + CFA (1 : 0), and rVV-CTB::GAD + CFA (1 : 10) treatment group and naive (PBS) mice. (b) 4-5 mice from each experimental groups were euthanized and the synthesis of cytokines and chemokines in GAD-activated splenocytes was analysed. Means ± standard errors are shown for 4-5 mice/groups. Differences between ages of the treated groups of mice compared to PBS are statistically significant (one-way Anova, *P* < 0.05). Differences between groups in blood glucose and data of percentage of insulitis are statistically insignificant (one-way Anova, *P* > 0.05).

**Figure 5:**
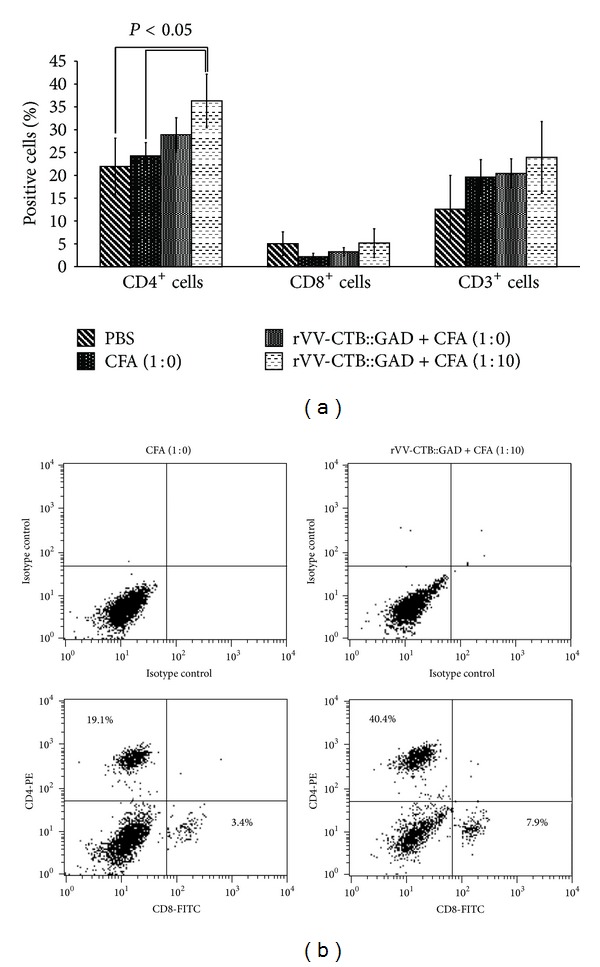
Analyses of splenocyte T-cell subsets. (a) Percentage of the CD3^+^, CD4^+^, and CD8^+^ T-cell subsets in splenocytes of experimental mice measured by flow cytometry. The splenocytes of the hyperglycemic PBS (*n* = 4, median and average blood glucose 33 mmol/L, median age 21 wks, average 21.25 wks of age), euglycemic CFA (1 : 0) (*n* = 4 median 8.05 mmol/L, average 7.88 mmol/L, median/average 119 wks), rVV-CTB::GAD + CFA (1 : 0) (*n* = 3, median 7.7 mmol/L, average 7.77 mmol/L, median/average 116 wks), and rVV-CTB::GAD + CFA (1 : 10) (*n* = 3 median 6.8 mmol/L average 6.43 mmol/L, median 119 wks, average 117 wks) treated mice were analyzed after stimulation with GAD65. Differences between percentage of the CD4^+^ T-cell subsets of the gated cell population of the mice inoculated with rVV-CTB::GAD + CFA (1 : 10) compared to PBS and CFA (1 : 0) are statistically significant (*P* = 0.027 and 0.018, resp.; one-way Anova). (b) Example of the relationship between the percentage of the CD4^+^ and CD8^+^ T-cell subsets of the gated cell population of the mice inoculated with CFA (1 : 0) and rVV-CTB::GAD + CFA (1 : 10). Cells were stained with antibodies for CD4 and CD8, as described in Section 2, and analyzed by flow cytometry. (The percentages of cells in quadrant, are shown relative to gated cells. Representative data from one mouse is displayed.)

For analyses of splenocytes, during the experiments 4-5 mice from four groups, control (PBS), and three treatment groups, were euthanized. The synthesis of cytokines and chemokines in GAD65-activated splenocytes of each mouse was analyzed using Mouse Cytokine Array Panel A kit of the Proteome Profiler Array (R&D Systems, Inc., Minneapolis, MN), as described in methods. Mean age, blood glucose levels section and degree of insulitis development in each group are presented in Figure 4(b). The control group was characterized by much higher blood glucose levels (mean value = 28.38 mmol/L) and younger mean age (40.8 wks). Most of the surviving mice in the experimental groups had blood glucose sugar levels within the range of 12.9–14.07 mmol/L (mean values) and older mean age (113–114.5 wks). Although in many cases histological analyses showed a high percentage of insulitis (Figure 4(b)). In all vaccinated groups the synthesis of IL-1*α* (IL-1F1), IL-1*β* (IL-1F2), IL-3, IL-4, IL-7, IL-13, TNF-*α* (TNFSF1A), IL-1ra (IL-1F3), KC, JE (CCL12), MIP-1*α* (CCL3), and MIP-2 was reduced by 5.5–40% in comparison with the PBS control mice. In addition, in 2 of 3 treated groups the synthesis of IL-2, IP-10 (CXCL10), TIMP-1, sICAM-1 (CD54), and MIG (CXCL9) was reduced by 9–27%. In contrast, synthesis of IL-16 was higher by 17.5–19.3% in 2 of 3 treated animal groups as compared to PBS control. The level of the synthesis of IL-10 was reduced by 22.8 only in mice treated with rVV-CTB::GAD + CFA (1 : 10), but the level of IFN-*γ* was higher by 10.4%, or was reduced in other experimental groups by 9–19.6% in comparison with the PBS control mice. Synthesis of RANTES (CCL5) was reduced by 16.7% only in mice treated with rVV-CTB::GAD + CFA (1 : 0). Other cytokines/chemokines (data not shown) in other experimental groups were similar (Figure 4(a)). The level of cytokines IL-12, IL-5, and IL-6 was found to be approximately at background (data not shown). Analyses of the relationship between the percentage of the CD3^+^, CD4^+^, and CD8^+^ cells of splenocytes show a low presence of CD8^+^ cells as compared to CD3^+^ and CD4^+^ lymphocyte subsets (Figures 5(a) and 5(b)). There were significant differences between the percentage of CD4^+^ T-cells in the gated lymphocyte population from mice inoculated with rVV-CTB::GAD + CFA (1 : 10) as compared to PBS and CFA (1 : 0) (*P* = 0.027 and 0.018, resp.; one-way Anova). However, there were no significant differences between the groups in the pattern of other T-cell subsets.

**Figure 6:**
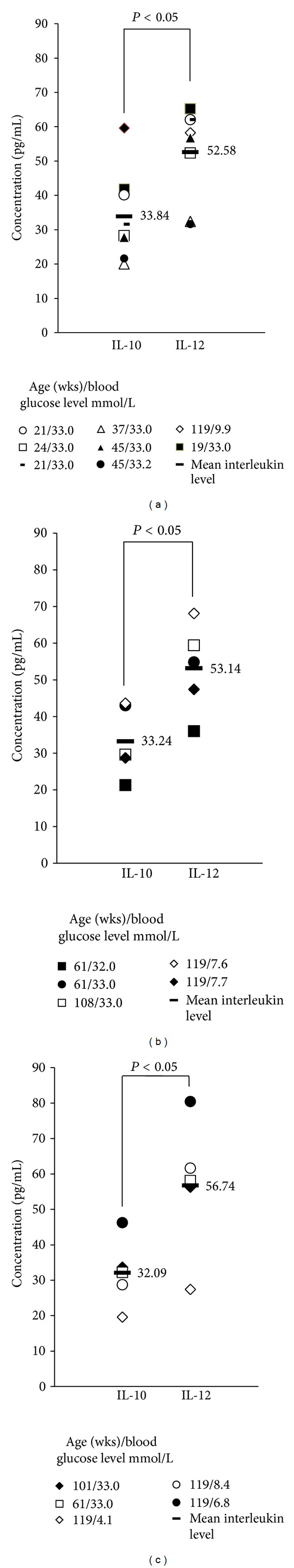
Detection of IL-10 and IL-12 in NOD mouse serum. (a) Secreted cytokines IL-10 and IL-12 in serum samples of the naive, (b) rVV-CTB::GAD + CFA (1 : 0), and (c) rVV-CTB::GAD + CFA (1 : 10) groups. Ages and blood glucose levels of individual mice are shown in columns on the right side.

Analyses of the levels of proinflammatory, type 1 cytokine IL-12 and immunosuppressive, type 2 cytokine IL-10 in sera of animals showed a similar pattern (*P* > 0.05) in three analyzed treatment groups (naive, rVV-CTB::GAD + CFA (1 : 0), and rVV-CTB::GAD + CFA (1 : 10) (Figures 6(a), 6(b), and 6(c))). The mean value of IL-12 was significantly (*P* < 0.05) higher (52–56 pg/mL) in all experimental groups as compared to IL-10 (32–38 pg/mL). We did not find any correlation between cytokine levels in serums or in the collected samples with the age and the health status of the animals (data not shown).

**Figure 7:**
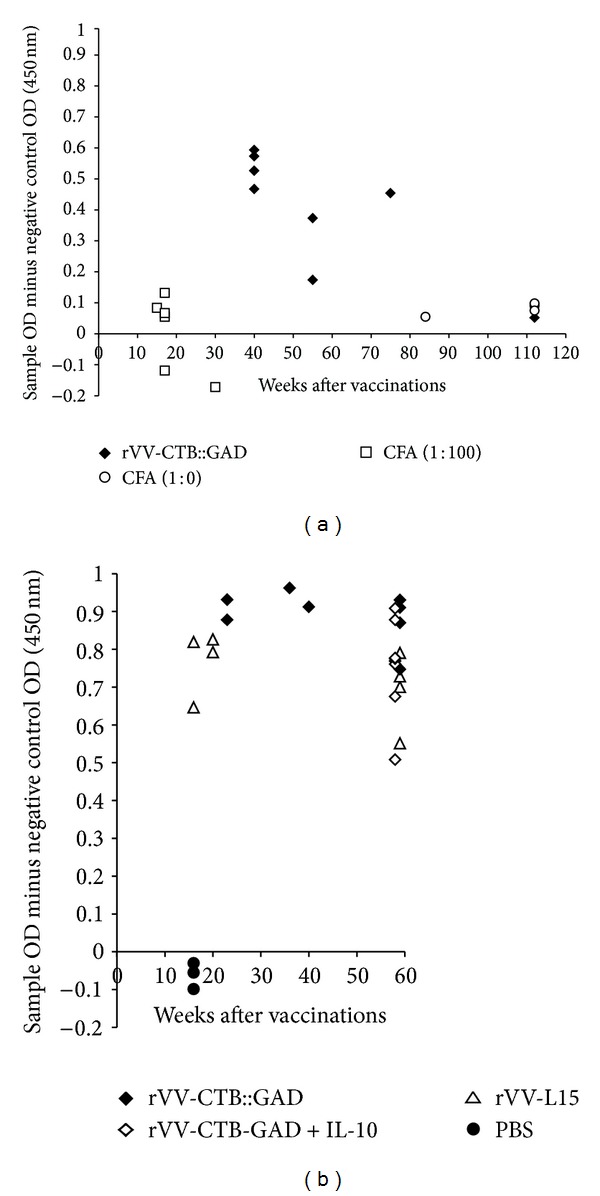
Vaccinia virus-specific humoral immune response in NOD mice. ((a) Exp. no. 1 and (b) Exp. no. 2). Determination of the VV-specific IgG was performed using indirect ELISA in two separate experiments. Naive (PBS), CFA (1 : 10), and CFA (1 : 100) mice served as controls for absence of VV-specific antibodies, while rVV-L15-treated [10] mice were controls for autoantigen-expressing virus rVV-CTB::GAD.

We also analyzed the dynamics of VV-specific antibody production in vaccinated and control animal groups throughout the experiment to determine whether vaccination had a deteriorating effect (*P* = 0.0004) on the immune system in general (Figures 7(a) and 7(b)). The results of these analyses show that antibody production in mice inoculated with the control recombinant virus rVV-L15 and recombinant virus-vaccine rVV-CTB::GAD was similar and remained high over a long period of time, up to 60–80 wks of age.

## 4. Discussion

A multifunctional approach of systemic coinoculation of juvenile NOD mice with both rVV-CTB::GAD and CFA demonstrated a marked, synergistic decrease in hyperglycemia and pancreatic islet inflammation in comparison with the PBS control. The three treatment groups with the highest level of beneficial outcome demonstrate a gradual increase in hyperglycemia from week 13 through 61 weeks of age. Thereafter, the levels of hyperglycemia did not change significantly in these groups. In the two most effectively immunosuppressed groups, rVV-CTB::GAD + CFA (1 : 0) and rVV-CTB::GAD + CFA (1 : 10), immunological homeostasis was maintained through 29 wks and 43 wks, respectively. Thereafter, rapid increases in hyperglycemia in these groups were observed, suggesting the length of vaccine experiments with NOD mice can be followed to at least 60 wks of age. In many laboratories evaluation of T1D vaccine efficacy in NOD mice was limited for less than 30 weeks, providing less informative results. Based on analysis of this long-term study thus dramatic changes in diabetes symptoms in vaccine treated animal groups may be observed in animals between 30 and 63 weeks of age.

Overall, our experimental results clearly indicate that combinatorial vaccination with either tricomponent vaccines, rVV-CTB::GAD plus CFA complete (1 : 0) or diluted (1 : 10), results in a dramatic reduction in diabetes onset in NOD mice. In future animal studies we will investigate the therapeutic effects of the vaccine in NOD mice when the vaccine is applied during later stages of diabetes development, for example, 20–40 wks of age. These data may provide clues for resolving the important question of whether the CTB-GAD + CFA vaccine strategy can prevent T1D in prediabetic children more effectively than in children diagnosed with new-onset disease.

Immunotherapy with major islet *β*-cell antigens such as insulin, glutamic acid decarboxylase (GAD), or heat shock protein (hsp60), with or without immunomodulators, was shown to interfere with or prevent T1D onset [13–17]. Oral delivery of CTB conjugated with specific autoantigens was shown to protect mice against several Th1 cell-mediated autoimmune diseases including autoimmune encephalomyelitis [18], autoimmune chondritis [19], and uveitis [20]. Further, oral delivery of CTB-autoantigen conjugates were shown to suppress diabetes insulitis and hyperglycemia in NOD mice and several other animal autoimmune diabetes models [21, 22]. These experimental results was associated with a reduction in inflammatory cytokine, IFN-*γ* production, and Tr1 regulatory T-cell migration into pancreatic islets [14, 23].

Previously, CFA and the closely related bacillus Calmette-Guerin (BCG), containing attenuated strain of *M. bovis* vaccines, were shown to modulate the development of T1D in animal models [24, 25]. Our experimental data confirm these previous experiments showing beneficial effects (60%) of CFA (1 : 0) and CFA (1 : 10) alone for prevention of T1D in NOD mice, which lasted until 59 and 37 weeks of age, respectively. An early study showed that the mycobacterial component of BCG serves as an immune potentiator of lymphocytes via TLR-mediated maturation of dendritic cells [26]. Administration of CFA was shown to prevent diabetes onset and to reduce the levels of insulitis in NOD mice [11]. In the KK-Ay mice studies, CFA vaccine provided controversial results [27]. In humans, BCG vaccination of T1D patients resulted in also conflicting data [25, 28]. Noteworthy, in currently ongoing clinical trials, D.L. Faustman expects that BCG will eliminate a population of disease-causing cells in T1D patients [29].

Immunotherapy with CFA was shown to be effective in preventing the spontaneous onset of autoimmune diabetes and in the restoration of self-tolerance to islet autoantigens [30, 31]. The protective effects of CFA were suggested to be mediated through the downregulation of autoreactive CTLs and the stimulation of NK cells. Most recent data however, demonstrate that CFA treatment ameliorates autoimmunity in NOD mice by up-regulating CD4^+^ CD25^+^ Foxp3^+^ regulatory T-cells in pancreatic lymph nodes and for increasing TGF-*β*1 production [32], in spite of the fact that altered frequencies of peripheral CD4^+^ CD25^+^ Foxp3^+^ regulatory T-cells were not yet shown to be specifically associated with type 1 diabetes [33].

Molecular mechanisms involved in autoantigen- and adjuvant-dependant immune suppression detected in NOD mice remain to be further elucidated. Evidence is available that CFA containing heat-inactivated *M. tuberculosis* cells induces T-cell-mediated immune responses, antibody production, and activation of the innate immune system [34]. Heat-shock protein-specific regulatory T-cells induced by mycobacteria may also contribute to CFA-induced suppression of diabetes [24]. Mycobacterial antigens may be presented to T-cells by different APC cells captured and presented by DC in NOD mice, sequestering capture and presentation of autoantigens. Protection may be attributable to an increase of antigen-presenting ability through maturation of DC and further that the mycobacterial cell wall skeleton is an essential adjuvant factor in CFA [26]. Complete Freund's Adjuvant was also suggested to induce clonal energy in effector cells that cause beta cell destruction [35].

An important issue linked to the adjuvants discussed here is their safety for human vaccine applications. Serendipitously, intravesical BCG was shown to be one of the most successful forms of immunotherapy in the treatment of human bladder cancer [36]. However, harmful effects, such as cystitis, dysuria, and haematuria, are often the main reason for discontinuing therapeutic treatment in many human studies. However, low-doses of BCG reduced these side effects without compromising therapeutic efficacy [37]. CFA was also the adjuvant of choice for immunization in laboratory animals for many decades. However, CFA has been associated with several local and systemic pathologies, including skin lesions and pneumonia [38]. Thus, CFA may cause significant side effects in humans, and lower doses may likely improve the safety features of the vaccine. In future studies, more precise dosage and frequency of CFA application will be established to allow the vaccine to be equally effective as well as safe for human vaccination applications.

Insulitis is thought to be associated with increased expression of proinflammatory cytokines (IL-1, TNF-*α*, and IFN-*α*) and type 1 cytokines IFN-*γ*, TNF-*β*, IL-2, IL-12 [39]. Lines of evidences suggest a role for chemokines in the pathogenesis of diabetes as well [40, 41]. Although previously we demonstrated that a combination of rVV-CTB::GAD and recombinant VV expressing the antiinflammatory cytokine IL-10 was effective in preventing the onset of T1D onset in NOD mice [10], we have not observed a significant increase in the synthesis of immunosuppressive IL-10 and IL-4 in any treatment groups of animals, as compared to PBS control. This experimental result is in agreement with a recent study of other authors [42]. In the sera of analyzed mice belonging to different treatment groups, we did not find changes in secreted IL-10 and IL-12 levels as well. Differences in the experimental results may be explained by difference in the environments where the individual treatments were performed. Our data confirm that multiple cytokines appear to participate in the autoimmune response that leads to *β*-cell destruction, and that deletion of a single “pathogenic” cytokine may not be sufficient to completely prevent diabetes development [39]. Although CD4^+^ and CD8^+^ T-cells are considered to be the primary mediators of *β*-cell destruction in NOD mice, our analyses of the relationship between the percentages of the CD3^+^, CD4^+^, and CD8^+^ cells in splenocytes did not confirm this notion.

To determine whether vaccine therapy may impair normal immune function, immunity to foreign antigens was assessed in NOD mice following diabetes remission. Analyses of VV-specific humoral immune response following vaccination showed that the levels of specific antibody production in mice vaccinated with the control recombinant virus rVV-L15 or the recombinant virus-vaccine rVV-CTB::GAD were high and remained high during an extended period of time (60–80 wks of age). Thus, immunological tolerance against T1D autoantigens induced by our vaccine does not reduce or alter normal immune responses against foreign antigens (Figures 7(a) and 7(b)). From a safety perspective it is important to note that inoculation of NOD mice with the most effective T1D vaccine formulation rVV-CTB::GAD + IL-10 [10] did not impair the anti-VV humoral immunity of the vaccinated mice (Figure 7(a)).

Certain viruses, for example, EMC-D and KRV, were shown to be associated with the development of T1D in several animal models and in humans [5]. Other studies on infection with pathogens showed that certain viruses may have an opposite effect by ameliorating T1D disease in diabetic mice [43]. These earlier data prompted us [9] and others [44] to study prevention of T1D using vaccinia viruses for delivery of genes encoding islet autoantigens. Jun et al. published experimental data claiming that VV expressing GAD may partially prevent diabetes in NOD mice [44]. Recombinant vaccinia virus experiments performed in our laboratory detected induction of an antiviral humoral immune response within 2 weeks following VV infection but did not show an effective VV-mediated or VV-GAD-mediated reduction of diabetes progression in NOD mice [10].

Vaccinia virus was shown to be a relatively safe and attractive virus vehicle for transgene delivery into a variety of mammals and humans for vaccination against infectious diseases [45]. To reduce potential side effects of live attenuated VV vaccines in immunocompromised individuals, we chose to use as a vaccine delivery vehicle the Lister vaccine strain of VV, which can be further attenuated by genetic manipulation [45]. Splenocytes from mock-infected mice secreted high levels of IFN-*γ* [10], whereas splenocytes isolated from mice inoculated with control VV secreted low to undetectable levels of the inflammatory cytokine IFN-*γ*. This result is in agreement with previous findings that VV infection of dendritic cells resulted in antigen-presenting cells that did not secrete inflammatory cytokines or initiate T-cell activation [46]. In contrast, VV expressing fused autoantigens, like insulin (rVV-CTB::INS) or GAD (rVV-CTB::GAD), could provide significant or moderate protection against development of T1D in NOD mice [9].

Here we demonstrated that systemic delivery of rVV expressing the immunostimulated autoantigen CTB::GAD, in combination with a normal or a 10-fold reduction in the dose of CFA, can confer a synergistic protective effect against diabetes onset in NOD mice. Further optimization of vaccine dosage may lead to more complete and safer protection of prediabetic NOD mice and humans against the progression of insulitis and hyperglycemia. An additional important goal of this study was to explore the duration of vaccine therapeutic effects. We found the majority of CTB::GAD + CFA-treated (1 : 0 and 1 : 10) mice remained euglycemic for more than a year up to 61–105 weeks of age, whereas most control mice developed hyperglycemia by 31 weeks of age. The levels of cytokines/chemokines were somewhat lower in the vaccinated mice, although the humoral immune system did not show signs of impairment. To better understand molecular mechanisms underlying the development of T1DM, therapeutic effects of this vaccination strategy will be assessed initially in NOD mice that have developed insulitis and hyperglycemia by 15–20 weeks of age. Moreover, the vaccine investigated in this study could be supplemented in the future with an additional viral construct expressing proinsulin (rVV-CTB::INS), which we demonstrated earlier to provide dramatic suppression of new diabetes onset in NOD mice [9]. The effectiveness of this multicomponent strategy for arresting or reversing the progression of insulitis and hyperglycemia in diabetic patients remains to be determined. Vaccinia virus-delivered immunomodulated autoantigens and CFA may provide continuous suppression of diabetes inflammation, thereby establishing an alternative approach for repopulation of the pancreas with “beta-like” insulin secreting cells, which could establish an effective and durable interventional therapy for restoration of euglycemia and immunological homeostasis in the large population of patients currently suffering from type 1 diabetes.

## Figures and Tables

**Table 1:** NOD mouse treatment groups for rVV-mediated suppression of hyperglycemia.

Groups	Treatment (i.p. and s.c. inoculation)	PFU (rVV)	Age at injection
1 (*n* = 10)	PBS (naive)	0	5 and 7 wks
2 (*n* = 10)	CFA (1 : 0)	0	5 and 7 wks
3 (*n* = 10)	CFA (1 : 10)	0	5 and 7 wks
4 (*n* = 10)	CFA (1 : 100)	0	5 and 7 wks
5 (*n* = 10)	rVV-CTB::GAD + CFA (1 : 0)	2 × (5 × 10^7^)	5 and 7 wks
6 (*n* = 10)	rVV-CTB::GAD + CFA (1 : 10)	2 × (5 × 10^7^)	5 and 7 wks
7 (*n* = 10)	rVV-CTB::GAD + CFA (1 : 100)	2 × (5 × 10^7^)	5 and 7 wks
8 (*n* = 10)	rVV-CTB::GAD	2 × (5 × 10^7^)	5 and 7 wks

**Table 2:** Analysis of hyperglycemia development.

	Treatment	PBS (naive)	CFA (1 : 0)	CFA (1 : 10)	CFA (1 : 100)	rVV-CTB::GAD + CFA (1 : 0)	rVV-CTB::GAD + CFA (1 : 10)	rVV-CTB::GAD + CFA (1 : 100)	rVV-CTB::GAD
Groups		1	2	3	4	5	6	7	8
		*P* values/weeks*							

		Incidence of diabetes							
1	PBS (naive)		**<0.01**/17–59 wks	≥0.05/19 wks and 31–37 wks	≥0.05/none	**<0.01**/ 17–105 wks	**<0.001**/ 17–61 wks	**<0.01**/ 17–51 wks	**<0.01**/ 17–43 wks
2	CFA (1 : 0)	**<0.001**		≥0.05/ none	**<0.01**/ 21–35 wks	≥0.05/ none	≥0.05/ 37–43 wks	≥0.05/ none	≥0.05/ none
3	CFA (1 : 10)	**<0.05**	**<0.001**		≥0.05/ 33 wks	**<0.01**/ 39–105 wks	**<0.01**/ 35–59 wks and 93–99 wks	≥0.05/ 39 wks	≥0.05/ 39 wks
4	CFA (1 : 100)	≥0.05	**<0.001**	≥0.05		**<0.01**/ 21–105 wks	**<0.01**/ 21–61 wks	**<0.05**/ 21–43 wks	**<0.01**/ 21–43 wks
5	rVV-CTB::GAD + CFA (1 : 0)	**<0.001**	**<0.05**	**<0.001**	**<0.001**		≥0.05/ none	≥0.05/ 103 wks	≥0.05/ 103 wks
6	rVV-CTB::GAD + CFA (1 : 10)	**<0.001**	**<0.05**	**<0.001**	**<0.001**	≥0.05		≥0.05/ 43 wks	≥0.05/ 43 wks
7	rVV-CTB::GAD + CFA (1 : 100)	**<0.001**	**<0.05**	**<0.001**	**<0.001**	**<0.001**	**<0.001**		≥0.05/ none
8	rVV-CTB::GAD	**<0.001**	**<0.001**	**<0.001**	**<0.001**	**<0.001**	**<0.001**	≥0.05	
		Blood glucose							

*P value for each pair of groups was determined using two-tailed Mann-Whitney *U* value. Test for equality of two proportions was used to determine those weeks when statistically significant differences (*Z* values between −1.96 and +1.96) were detected.
